# Down‐sampling template curve to accelerate LDDMM‐curve with application to shape analysis of the corpus callosum

**DOI:** 10.1049/htl2.12011

**Published:** 2021-05-02

**Authors:** Weikai Huang, Xiaoying Tang

**Affiliations:** ^1^ Department of Electrical and Electronic Engineering Southern University of Science and Technology Shenzhen Guangdong China

## Abstract

Large deformation diffeomorphic metric mapping for curve (LDDMM‐curve) has been widely used in deformation based statistical shape analysis of the mid‐sagittal corpus callosum. A main limitation of LDDMM‐curve is that it is time‐consuming and computationally complex. In this study, down‐sampling strategies for accelerating LDDMM‐curve are investigated and tested on two large datasets, one on Alzheimer's disease (155 Alzheimer's disease, 325 mild cognitive impairment and 185 healthy controls) and the other on first‐episode schizophrenia (92 first‐episode schizophrenia and 106 healthy controls). For both datasets a variety of down‐sampling factors are tested in terms of registration accuracy, registration speed, and most importantly disease‐related patterns. Experimental results revealed that down‐sampling template curve by a factor of 2 can significantly reduce the running time of LDDMM‐curve without sacrificing the registration accuracy. Also, the disease‐induced patterns, or more specifically the group comparison results, were almost identical before and after down‐sampling. It is also shown that there was no need to down‐sample the target population curves but only the single template curve of the study of interest. Comprehensive analyses were conducted.

## 1 INTRODUCTION

Large deformation diffeomorphic metric mapping (LDDMM) is one of the state‐of‐the‐art anatomical manifold matching algorithms that has been successfully applied to characterising localised morphometrics of different brain structures of interest in various neurodegenerative disorders [1, 2, 3, 4]. This general registration framework can work for manifolds of different dimensions such as landmarks, curves, surfaces, and dense images.

LDDMM for curve (LDDMM‐curve) [5] has been applied to statistical shape analyses of various 2D brain regions of interest (ROIs) [6, 7], such as the 2D mid‐sagittal corpus callosum (CC). The CC is the largest white matter structure in the human brain and the largest commissural fibre bundle connecting the two cerebral hemispheres, and thus it has been the research object of interest in various neurodegenerative disorders such as Alzheimer's disease (AD) [8], Huntington's disease [9] and schizophrenia [10].

In LDDMM‐curve based statistical shape analysis pipeline, the LDDMM‐curve registrations between a template curve and all target population curves are most time‐consuming which usually takes hours or even days especially when the sample size of the population of interest is large. Therefore, accelerating the LDDMM‐curve registration process to reduce the computational cost is very important both scientifically and practically, and thus is the problem of interest of this work. Previous studies on accelerating LDDMM has been relying on GPU‐based parallel computing [11, 12, 13, 14]. In addition to GPU‐based acceleration, Wu et al. [15] proposed a new optimisation strategy to accelerate the LDDMM registration process, but only for registering images. There have also been other types of efforts in this respect. For example, Hernandez [16] formulated the problem in the space of band‐limited vector fields and combined with semi‐Lagrangian integration with respect to three variants of band‐limited PDE‐constrained LDDMM to further boost the computational efficiency.

Another potential solution is to decrease the discretised sample points when representing anatomical manifolds of interest. For example, in LDDMM‐image, down‐sampling the images of interest to have less voxels, in LDDMM‐surface, down‐sampling the surfaces of interest to have less vertices, and in LDDMM‐curve, down‐sampling the curves of interest to have less points will surely reduce the computation time of LDDMM. However, there may be potential issues. Firstly, the registration accuracy may be affected. Secondly, the study conclusions may be altered, especially in disease‐related abnormality identification and quantification. As such, it is of great importance to investigate a proper down‐sampling strategy, which is the essential motivation of this study. Our goal is to figure out the most appropriate down‐sampling strategy for LDDMM‐curve when applied to CC, aiming at a good balance of registration accuracy, computation time, as well as disease‐related abnormality patterns.

In this study, we take two brain disorders for example, with one being AD and the other one being first‐episode schizophrenia (FES). Two large sets of T1‐weighted magnetic resonance images (MRIs) are employed to analyse the 2D mid‐sagittal CC curve. For AD, the sample size is 665, involving 185 matched healthy control (HC) participants, 325 mild cognitive impairment (MCI) patients and 155 AD patients. For FES, the sample size is 198, involving 106 matched HC participants and 92 FES patients. A total of 4 down‐sampling rates have been investigated, being respective 2, 3, 4 and 5. Both quantitative and qualitative analyses are performed, in terms of computational time, registration accuracy, as well as control‐versus‐disease group differences.

To conclude, the main contributions of this paper are twofold:

• We propose a simple but highly effective down‐sampling strategy for LDDMM‐curve acceleration.

• We extensively test and successfully validate the proposed approach on two large T1‐weighted structural MRI datasets focusing on different brain diseases.

**FIGURE 1:**
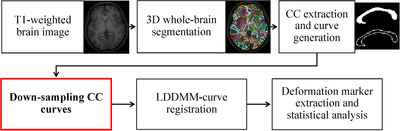
Block diagram of the entire pipeline

The rest of this paper is organised as follows. In Section 2, the two T1‐weighted MRI datasets, CC curve generation steps, down‐sampling strategies, LDDMM‐curve framework, and statistical shape analysis and evaluations are introduced. The block diagram of the entire pipeline is shown in Figure 1. In Section 3, experimental results are presented. Finally, Section 4 concludes the paper.

## 2 MATERIALS AND METHODS

### 2.1 Datasets

In this study we utilised two T1‐weighted structural MRI datasets. The first dataset was obtained from the Alzheimer's Disease Neuroimaging Initiative (ADNI) study (adni.loni.usc.edu) involving 185 HC participants (95 females and 90 males), 325 MCI patients (119 females and 206 males), and 155 AD patients (74 females and 81 males).

Participants in the second dataset were recruited from the First Affiliated Hospital of Shenzhen University and a total of 198 participants were involved, including 92 FES patients (50 females and 42 males) and 106 HCs (47 females and 59 males). Informed consents were obtained from all participants or family relatives, as approved by the Ethics Committee at the First Affiliated Hospital of Shenzhen University. All structural MRI data were acquired from a Siemens Trio Tim 3T scanner and were visually examined for data quality control.

### 2.2 CC curve generation

An initial step in this study was to extract the mid‐sagittal CC curve from each whole‐brain T1‐weighted 3D volume image. Please note CC consists of the genu (anterior), body and splenium (posterior) , namely gCC, bCC and sCC. We first rigidly aligned each image to the MNI space [17] and then utilised a validated automatic segmentation algorithm [18] to get its 3D whole‐brain segmentation in the MNI space. Each segmentation result was visually inspected and manually corrected in case of automated segmentation errors. We extracted the binary segmentation results of gCC, sCC and bCC and combined them to form the 3D CC segmentation and took out the 2D mid‐sagittal CC slice. In the MNI space, there are a total of 181 sagittal slices and thus the 2D mid‐sagittal one refers to the 91st slice. After obtaining a 2D mid‐sagittal CC slice, an edge detection algorithm [19] was applied to identify the boundary pixels and those pixels were arranged clockwisely to form a CC curve. For each dataset we identified a template curve whose area was closest to the mean area averaged across all curves in the corresponding dataset.

### 2.3 Down‐sampling CC curves

In this study, the range of the down‐sampling factors was set to be 2 to 5. In addition, because LDDMM‐curve was performed between a template curve and each target curve, we also analysed the difference between two down‐sampling schemes, with one being down‐sampling both the template curve and all target curves and the other one being down‐sampling the template curve only.

### 2.4 LDDMM‐curve and statistical shape analysis

For both datasets, each target curve was first rigidly aligned (rotation and translation) to the template curve to reduce the computation cost of subsequent LDDMM‐curve and to improve its registration accuracy. After that, LDDMM‐curve was used to get a diffeomorphic transformation mapping the template curve to the rigidly aligned target curve. LDDMM‐curve makes use of a dynamic flow of diffeomorphisms $\phi_{t}^{v}$ in the ambient space $R^{d}$ to build a correspondence between the template and the target [5]. The dynamic flow $\phi_{t}^{v}$ is parameterised by a time‐dependent velocity vector field, $v_{t}:R^{d}\to R^{d}$ for t∈[0,1], via the ordinary differential equation: $\frac{\partial \phi_{t}^{v}}{\partial t}=v_{t}\left(\phi_{t}^{v}\right)$ [5].

We have $\phi_{0}^{v}\left(x\right)=x$ and the consequent diffeomorphism is $\phi_{1}^{v}$ at the end time t=1. Assume the template curve is C and the target curve is S, the goal of LDDMM‐curve is to optimise $v_{t}$ so that the associated diffeomorphism $\phi_{1}^{v}$ can accurately map curve C to curve S. The energy function in LDDMM‐curve is

(1) $$J_{C,S}\left({\left(v_{t}\right)}_{t\in [0,1]}\right)≐\gamma \int_{1}^{0}{\left\|v_{t}\right\|}_{V}^{2}\mathrm{dt}+E\left(\phi_{1}^{v}\cdot C,S\right).$$

The first term in the energy function is a regularisation term guaranteeing the smoothness of the deformation field. The space V is a reproducing kernel Hilbert space which is also a space of smooth vector fields to guarantee that the transformations are diffeomorphic. The second term is a matching function that quantifies the distance between the deformed template curve $\phi_{1}^{v}\cdot C$ and the target curve S. More details regarding the LDDMM‐curve algorithm can be found in its original publication [5].

After obtaining the diffeomorphic transformation from LDDMM‐curve, at each point of the template curve we computed the Jacobian matrix $D_{\phi_{t}^{v}}$ and obtained a “deformation marker” $J=\text{det}(D_{\phi_{t}^{v}})$ (the determinant of the Jacobian matrix $D_{\phi_{t}^{v}}$). The value of the deformation marker J corresponds to the localised shape variation of each point on the template curve. Specifically, a value of J larger than 1 represents outward‐deformation of the target curve relative to the template curve at a local point, otherwise inward‐deformation. This deformation marker was then used in our statistical comparisons.

For each target curve s, $J_{k}\left(s\right)$ represents the deformation marker at point k of the template curve. The statistical analysis was conducted using the following linear model

(2) $$J_{k}\left(s\right)=\beta_{k,0}+\beta_{k,1}\gamma \left(s\right)+\sum_{\text{cov}}\alpha_{\text{cov}}X_{\text{cov}}\left(s\right)+\varepsilon_{k}\left(s\right).$$

In this model γ(s) is a binary group variable; γ(s) equals to 1 when the target curve s belongs to one group and 0 when the target curve s belongs to the other group. $X_{\text{cov}}\left(s\right)$ denotes the covariate information and we covaried for age, gender, and total intracranial volume in this study. $\varepsilon_{k}\left(s\right)$ represents a Gaussian noise variable. For each point k on the template curve, we tested the null hypothesis that $\beta_{k,1}=0$. We used Fisher's method of randomisation and permutation tests to quantify the statistical significance of the difference between two groups under comparison and the *p*‐values were corrected for multiple comparisons by controlling the family‐wise error rate (FWER) at a level of *p* ≤ 0.05. In the process of permutation tests, we generated 10,000 uniformly distributed random permutations by employing Monte Carlo simulations. We used $-\beta_{k,1}$ to denote the degree of group difference, so that a positive value represents atrophy in the second group relative to the first group whereas a negative value represents expansion.

### 2.5 Evaluations

To assess the computational performance of the down‐sampling strategy, for each down‐sampling factor, we divided the total registration time by the number of target curves as the average computational time (seconds per target curve). And we used the Dice score [20] between the target curve and the deformed template curve after LDDMM‐curve to quantify the registration accuracy. A larger Dice score indicates a higher registration accuracy.

For the purpose of evaluating the effect of down‐sampling exerted to the group shape comparison results, we up‐sampled the group difference results by the corresponding factor to ensure the number of curve points after up‐sampling is the same as that of the original results. An overall error was used to quantify the difference between the two results

(3) $$\text{Overall}\;\text{error}=\frac{\sum_{i=1}^{n}{\left|r\left(i\right)-r_{1}\left(i\right)\right|}^{2}}{\sum_{i=1}^{n}{\left(\left|r(i)\right|+\left|r_{1}\left(i\right)\right|\right)}^{2}}.$$

In this error expression r= original result, $r_{1}=$ result obtained after down‐sampling curves which was then up‐sampled, n= the total vertex number. The overall error value ranged in [0, 1].

## 3 RESULTS

### 3.1 Registration performance

In this study, for both datasets, we analysed LDDMM‐curve's registration performance in terms of running time and registration accuracy for each of the down‐sampling factors from 2 to 5 as listed in Tables 1 and 2. The *p*‐values obtained from paired Student's *t*‐tests on the Dice score are also provided. Obviously, for the same down‐sampling factor, down‐sampling template curves only is similar to down‐sampling both template and target curves in terms of computational time. However, the registration accuracy is higher when only down‐sampling the template curves. Across all four down‐sampling factors, for both datasets, 2 is the best balancing computational efficiency and registration accuracy. Clearly, the registration accuracy decreases significantly when down‐sampling the template curve by each of the other three factors, with a *p*‐value being smaller than 0.05.

**TABLE 1:** Registration performance results obtained on the first dataset

Down‐sampling factor	Running time (s/target curve)	Dice score	Dice *p*‐value
Original	18.5	0.89	/
2(template and target)	4.57	0.893	0.005
2(only template)	4.72	0.895	**0.074**
3(template and target)	2.19	0.887	3.43e‐12
3(only template)	2.29	0.891	3.57e‐5
4(template and target)	1.36	0.886	4.55e‐14
4(only template)	1.54	0.889	8.07e‐9
5(template and target)	1.03	0.876	1.94e‐39
5(only template)	1.20	0.884	3.35e‐13

Bold typesetting indicates no significance from Student's *t*‐test.

**TABLE 2:** Registration performance results obtained on the second dataset

Down‐sampling factor	Running time (s/target curve)	Dice score	Dice *p*‐value
Original	16.45	0.926	/
2(template and target)	4.52	0.922	0.020
2(only template)	4.57	0.923	**0.110**
3(template and target)	2.34	0.919	4.13e‐4
3(only template)	2.44	0.922	0.023
4(template and target)	1.41	0.917	1.60e‐6
4(only template)	1.52	0.920	**0.089**
5(template and target)	1.04	0.910	8.41e‐18
5(only template)	1.27	0.917	1.02e‐5

Bold typesetting indicates no significance from Student's *t*‐test.

### 3.2 Localised shape difference

To evaluate the influence of down‐sampling exerted to control‐versus‐disease shape group comparison results, we compared the localised shape difference results after down‐sampling with the original results, the overall error results of which are displayed in Table 3. Clearly, down‐sampling template curves only by a factor of 2 also yields the best result in terms of localised control‐versus‐disease shape group difference results, for both datasets.

**TABLE 3:** The overall error obtained from comparing the localised shape difference result under each down‐sampling factor and that of the original result on the first and second datasets

	HC vs. Disease group		
Down‐sampling factor	MCI	AD	FES
2(template and target)	3.12%	4.83%	4.28%
2(only template)	**3.15%**	**1.23%**	**3.95%**
3(template and target)	4.77%	2.15%	4.94%
3(only template)	4.70%	7.45%	4.70%
4(template and target)	9.26%	11.04%	5.54%
4(only template)	6.43%	7.08%	7.91%
5(template and target)	37.48%	7.00%	7.36%
5(only template)	7.24%	10.94%	8.00%

Bold typesetting suggests the best result.

**FIGURE 2:**
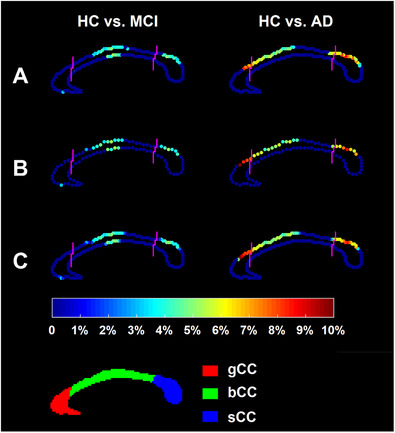
The localised shape analysis results of the first dataset. A demonstrates the original results, B demonstrates results after down‐sampling the template curve by a factor of 2 and C demonstrates up‐sampled results of B. The CC sub‐region definitions are illustrated at the bottom panel. The color bar denotes the degree of atrophy in the patient group relative to the control group

**FIGURE 3:**
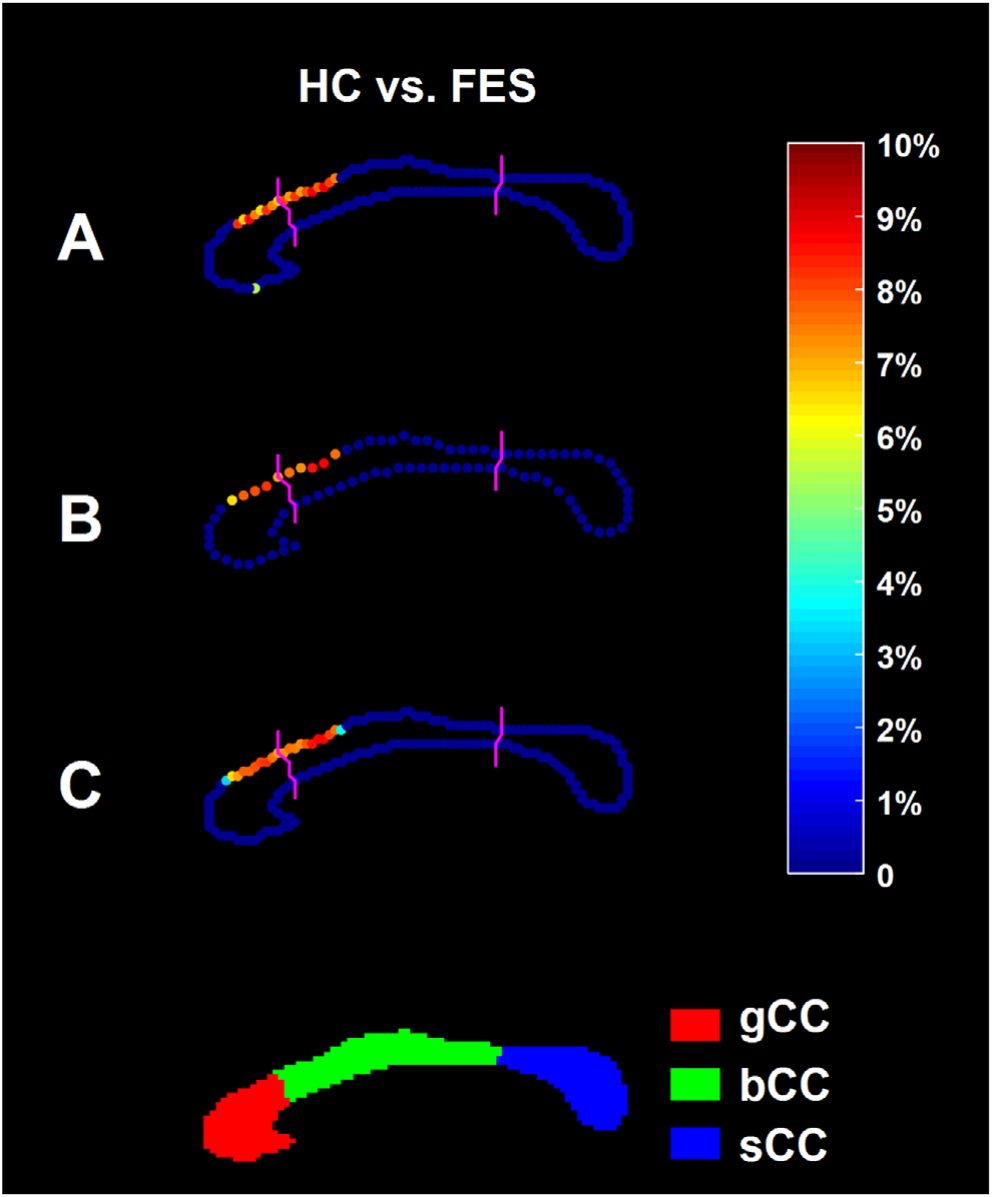
The localised shape analysis results of the second dataset. A demonstrates the original results, B demonstrates results after down‐sampling the template curve by a factor of 2 and C demonstrates up‐sampled results of B. The CC sub‐region definitions are illustrated at the bottom panel. The color bar denotes the degree of atrophy in the patient group relative to the control group

Figures 2 and 3 respectively demonstrate the localised shape difference results obtained from the first and the second dataset; specifically, the original localised shape comparison results and the corresponding results obtained from down‐sampling the template curves by a factor of 2. In those two figures, the points highlighted denote the ones whose localised shape characteristics differed significantly (after FWER correction) between the two groups under comparison, and the colour bar represents the degree of atrophy in the disease group relative to the control group. Obviously, the location and degree of atrophy of the results obtained from down‐sampling the template curves by a factor of 2 are quite close and almost identical to that of the original results. The *p*‐values obtained from the shape group comparison are also very similar before and after down‐sampling. Specifically, before and after down‐sampling, the *p*‐values are 0.0001 and 0.0002 for HC versus AD, 0.0018 and 0.0031 for HC versus MCI, and 0.0123 and 0.0145 for HC versus FES.

## 4 CONCLUSIONS

In this study, we comprehensively investigated a curve down‐sampling strategy to accelerate the LDDMM‐curve registration process. Existing acceleration strategies for LDDMM mainly rely on GPU based parallel computing [11, 12, 13, 14]. The primary advantage of the proposed down‐sampling method is that it is very easy to implement whereas GPU based parallel computing requires environment configuration which can be quite complex and is also very expensive. A potential downside of down‐sampling is that it may impair the registration accuracy and may also affect disease‐induced abnormality patterns, especially in localised shape analyses.

To find a suitable down‐sampling factor, for both datasets we analysed four different down‐sampling factors and for each down‐sampling factor we compared the results after down‐sampling and the original results. When we increased the down‐sampling factor, the running time decreased but the registration accuracy also decreased, and the error in terms of the localised shape group difference also increased. Jointly considering the computational efficiency, the registration accuracy, and the control‐versus‐disease pattern, down‐sampling by a factor of 2 worked the best. Also, it is worth being pointed out that there is no need to down‐sample both the template curve and the target curves but only the template curve. In other words, down‐sampling the single template curve by a factor of 2 can significantly improve the computational efficiency of LDDMM‐curve without sacrificing any of the registration accuracy nor the disease‐related abnormality pattern. This method can be also extended to other applications, such as LDDMM‐curve based facial expression recognition [21] and LDDMM‐curve based mitochondrial motion analysis [22].

According to our experimental results, we can draw a conclusion that down‐sampling the single template curve by a factor of 2 can accelerate LDDMM‐curve by about four times without sacrificing the accuracy nor the disease pattern. There are still limitations in this current study. For example, compared with GPU‐based parallel computing methods [11, 12, 13, 14], our acceleration approach is limited by the down‐sampling factor. And the effectiveness of the proposed pipeline with respect to manifolds of other dimensions, such as surfaces and dense images, is not established. Future research directions include two components: (1) Combining the proposed down‐sampling strategy with GPU‐based parallel computing may further enhance the computational efficiency, and is a research direction that is worthy of pursuing. (2) To enlarge the application scope of our proposed strategy, validation experiments on manifolds of higher dimensions including surfaces and images are needed. That will also be one of our future research plans.
